# Convergent multi-modular architecturefor adaptive learning in *Drosophila* and artificial intelligence

**DOI:** 10.1016/j.isci.2025.113799

**Published:** 2025-10-17

**Authors:** Liyuan Wang, Qian Li

**Affiliations:** 1 Department of Computer Science and Technology, Institute for AI, BNRist Center, THBI Lab, Tsinghua University, Beijing 100084, China; 2 School of Medicine, Shenzhen Campus of Sun Yat-sen University, Shenzhen 518107, China

**Keywords:** Artificial intelligence, Evolving intelligent system, Machine learning

## Abstract

Faced with dynamic, uncertain environments, a common goal of biological intelligence (BI) and artificial intelligence (AI) is to develop robust adaptive learning capabilities, despite different origins. Exploring shared mechanisms may uncover universal computational principles of information processing. In this perspective, we review recent studies of the *Drosophila* olfactory learning system, which exemplifies a hierarchical multi-modular architecture with specific connectivity patterns. Through an interdisciplinary comparison of anatomical and functional characteristics, we find that it reflects an elegant combination of two classical multi-modular methods in machine learning: ensemble learning (EL) and mixture-of-expert (MoE). This biological hierarchy incorporates the respective strengths of EL and MoE to improve adaptability and employ effective strategies to address their technical challenges, promoting generalization and alleviating interference on a continual basis. We further propose interdisciplinary research directions, such as developing bio-inspired machine learning models that reconcile EL and MoE, and conducting targeted biological experiments to dissect modular learning functions.

## Graphical abstract

**Figure:**
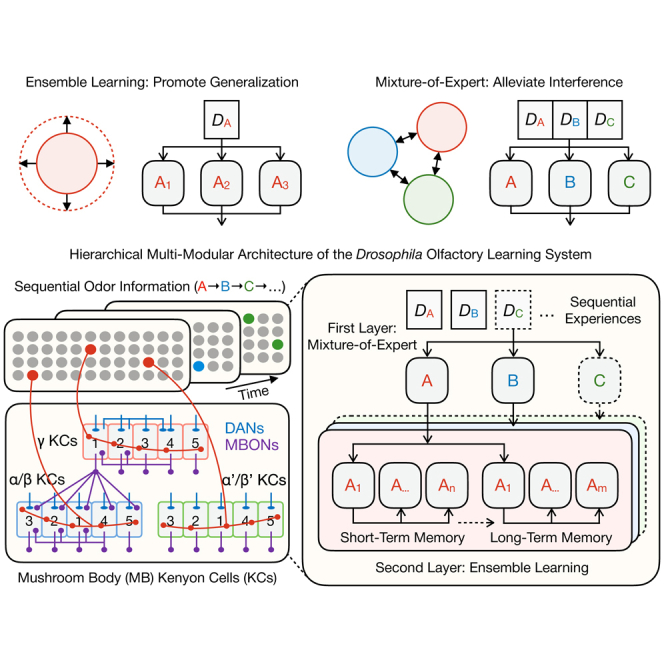

## Introduction

Biological intelligence (BI) and artificial intelligence (AI) emerge from fundamentally different origins, yet both must develop strong adaptive learning capabilities to function in dynamic, uncertain environments. Over the course of evolution, biological systems from invertebrates to humans have gradually developed effective strategies for adaptation, shaped by pressures of survival and reproduction. Their learning processes are embedded in neural architectures optimized not for precision or task-specific performance, but for robustness in noisy, ever-changing conditions. In contrast, artificial systems are engineered with explicit objectives, such as maximizing accuracy, efficiency, or scalability, typically within well-defined, static settings. Consequently, while BI emphasizes behavioral flexibility and resilience, AI often prioritizes performance relative to fixed benchmarks. This divergence highlights the importance of cross-disciplinary comparisons to understand how different systems achieve the common goal of continual adaptation.

**Figure 1:**
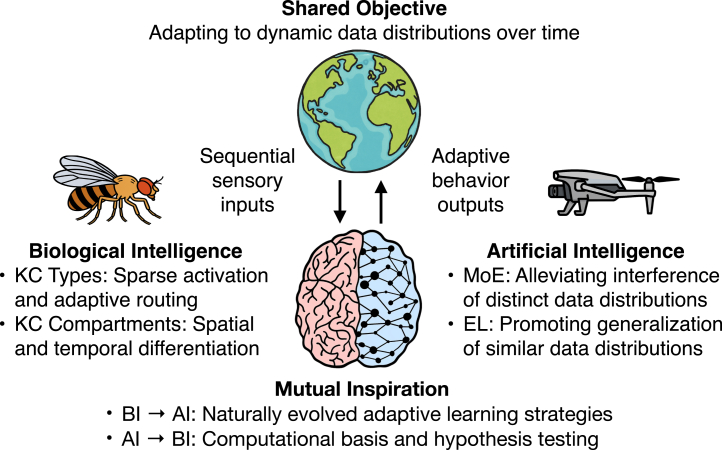
Bridging biological and artificial intelligence for adaptive learning Biological intelligence (BI) and artificial intelligence (AI) both aim to adapt to changing data distributions from the real world. The emerging field of NeuroAI seeks to integrate its complementary strengths: BI offers naturally evolved architectures and learning strategies, while AI provides implementation frameworks for computational modeling and hypothesis testing.

The emerging field of NeuroAI^1^ offers a promising framework for bridging biological learning and machine learning by integrating their complementary strengths^2^^,^^3^^,^^4^ (Figure 1): biological learning systems provide naturally evolved architectures and adaptive mechanisms, while machine learning models enable scalable computation, hypothesis testing, and theoretical analysis. Since the 1970s, researchers have discovered many neurobiological mechanisms of learning and memory by leveraging the unique advantages of different model organisms, including *Aplysia*, *Drosophila*, and mice.^5^ Among these, the fruit fly *Drosophila* has struck an excellent balance between the clarity of brain networks and the complexity of behavioral responses. In recent years, rapid progress has been made in studying the neurobiological mechanisms of olfactory associative learning and memory in *Drosophila*, especially for its functional neural circuits and synapse-level connectomes, demonstrating a sophisticated architecture centered around the mushroom body (MB).^6^^,^^7^^,^^8^^,^^9^^,^^10^^,^^11^

As the center for olfactory associative learning, the MB must, on one hand, possess the ability to discriminate odors, and on the other hand, be capable of flexibly assigning punishment or reward values to specific odors based on sequential experiences. Research on the structural and functional neural circuits related to the former (odor discrimination) has already provided valuable insights into multiple computational problems, such as locality-sensitive hashing,^12^ novelty detection,^13^ and count sketch.^14^ Remarkably, when artificial neural networks are trained to classify odor identity, they evolve similar features to the *Drosophila*’s MB circuit, suggesting a convergence of their computational principles.^15^ However, with regard to the latter (flexible value assignment over time), whether the MB and artificial systems also share convergent architectural strategies in the context of sequential learning tasks remains an open question. Beyond olfactory learning and memory, the MB also integrates internal states and contributes to value-based decision-making,^16^ further underscoring its role in adaptive behaviors.

Structurally, the *Drosophila* MB exhibits a hierarchical multi-modular architecture with specific connectivity patterns, enabling robust behavioral adaptation to external changes. Comparably, multi-modular architectures have also been widely adopted in machine learning to enhance adaptability, with ensemble learning (EL)^17^ and mixture-of-expert (MoE)^18^ being two classical representatives. Although both methods combine multiple individual models, EL aims to promote generalization for learning the same data distribution, while MoE aims to alleviate interference for learning distinct data distributions. However, they face their own technical challenges and have inconsistent learning objectives when working together, especially in practical applications that require accumulating knowledge and expanding model capacity over time.

Through an interdisciplinary comparison of anatomical and functional characteristics, we find that the hierarchical multi-modular architecture in *Drosophila* appears to be an elegant combination of EL and MoE: different hierarchical layers reconcile their distinct learning objectives, while incorporating effective strategies to address associated technical challenges. We further explore how these neurobiological mechanisms could inspire the design of more adaptive machine learning models, and how these computational counterparts might extend the understanding of biological learning, offering innovative perspectives for advancing BI and AI together. In particular, the recent availability of the *Drosophila* whole-brain connectome data^11^ has provided an unprecedented opportunity to further investigate how the MB-based learning system is integrated into the whole-brain network to fulfill its functions, as well as to compare the architectures of biological and artificial learning systems.

## Brain networks for olfactory learning and memory in *Drosophila*

Faced with unexplored environments, adult fruit flies innately approach food-related odors and avoid predator-related odors, suggesting that their brain networks encode certain forms of “prior knowledge”. However, as environmental conditions change, these innate odor-driven behaviors alone are insufficient for adaptation. To address this, *Drosophila* possesses an experience-dependent learning ability to assign reward or punishment values to specific odors.^19^^,^^20^ The olfactory learning and memory in *Drosophila* is served by its brain networks with functional differentiation across various levels, including brain regions, neuronal populations, and compartments.

**Figure 2:**
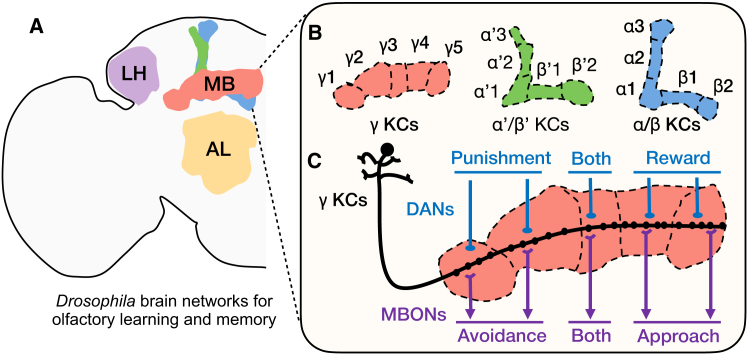
The *Drosophila* olfactory learning system (A) Main regions involved in olfactory learning and memory, including the antennal lobe (AL), the mushroom body (MB), and the lateral horn (LH). (B) Schematic of axon bundles of the three major types of Kenyon cells (KCs), including γ KCs, α′/β′ KCs, and α/β KCs. The axon bundles of each type of KCs can be further divided into five compartments by specific connections to distinct dopamine neurons (DANs) and MB output neurons (MBONs). (C) Schematic of γ KCs with the five γ compartments. Each compartment receives external olfactory information, modulated by DANs and output by MBONs.

At the level of brain regions, olfactory information is first processed in the antennal lobe (AL), and then transmitted via projection neurons (PNs) to two higher centers: the MB mentioned above and the lateral horn (LH)^21^ (Figure 2A). The MB is a well-known center for learning and memory,^22^ and the LH is mainly responsible for innate behaviors.^23^ Although recent studies suggest functional interactions between these two regions,^24^^,^^25^ our discussion focuses on the potential benefits and underlying mechanisms of the MB in adaptive learning.

At the level of neuronal populations, a given odor can sparsely activate approximately 5% of around 2000 MB intrinsic neurons, called the Kenyon cells (KCs), on each side of the brain.^7^ These sparse activation patterns enable odors to be characterized and differentiated. KC axons further divide the MB into five lobes, α, β, α′, β′, and γ. Based on their axonal morphology within the lobes (Figure 2B), KCs can be classified into three major types: γ KCs, α′/β′ KCs, and α/β KCs. Each type plays distinct roles in memory processing, reflecting a clear division of labor. Specifically, the γ KCs are primarily responsible for the formation and forgetting of short-term memory (STM)^26^^,^^27^^,^^28^^,^^29^; the α′/β′ KCs are important for memory stabilization^30^^,^^31^; and the α/β KCs play a role in different types of long-term memory (LTM) formation and retrieval.^32^^,^^33^^,^^34^^,^^35^^,^^36^^,^^37^

Each lobe can be further divided into compartments (Figure 2B). At the level of compartments, the axons of each major type of KCs can be further divided into five compartments, based on their specific connection patterns with dopaminergic neurons (DANs) and MB output neurons (MBONs).^6^ These compartments exhibit functional specialization in altering behaviors,^7^^,^^38^^,^^39^ representing another division of labor. Taking γ KCs as an example (Figure 2C), odor information can activate different MBONs via KC-to-MBON synapses. The activation of MBONs in distinct γ compartments leads to approach or avoidance behaviors depending on their associated valence. Odor-associated punishment or reward signals modulate MBON activity by reinforcing KC-to-MBON synaptic plasticity through DANs. Specifically, DANs innervating γ1-γ2 compartments convey punishment signals, those innervating γ4-γ5 convey reward signals, and γ3 exhibits a mixed role: inhibition of DANs in γ3 mediates reward, whereas activation can drive avoidance behavior.^40^ Together, the organization of the MB emerges as a hierarchical multi-modular architecture with sparsely activated KCs and functionally distinct compartments, which supports experience-dependent olfactory learning over time.

## Two classical multi-modular methods in machine learning

**Figure 3:**
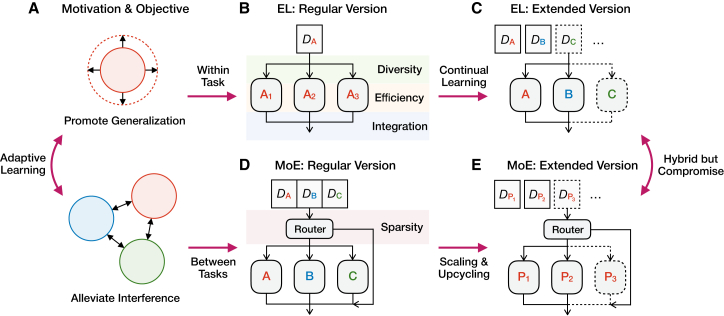
Two representative multi-modular methods in machine learning (A) Adaptive learning requires promoting generalization and alleviating interference on a continual basis. *D*_A_, *D*_B,_ and *D*_C_ denote the training data of tasks A, B, and C, respectively. (B) EL aims to promote the generalization of learning identical data distributions. It needs to properly induce diversity, improve efficiency, and perform the integration of multiple individual models. (C) The extension of EL to continual learning often involves learning each task with a specialized model, so as to prevent inter-task interference. (D) MoE aims to alleviate the interference of learning district data distributions. It incorporates a learnable or deterministic routing function for sparsely assigning inputs and integrating outputs. (E) MoE has been used extensively to scale up foundation models, improving generalization to various tasks while preserving resource efficiency. *D*_P_ denotes the pre-training data collected incrementally.

Similar to the *Drosophila* MB, multi-modular architectures are commonly used to enhance the adaptability of machine learning (Figure 3A). Among these, EL^17^ and MoE^18^ are the most classical representatives, each with its own motivations and technical challenges.^41^^,^^42^

EL was originally proposed to promote the generalization of learning the same data distribution through combining multiple individual models^17^^,^^43^ (Figure 3B). This strategy has proven to reduce generalization errors from training data to similar test data^43^ (corresponding to transferring odor-valence associations to similar odors in *Drosophila*), though it faces three major technical challenges in practice.^41^ The first is to induce diversity among these models. Since the observed data distributions are identical or similar, it is critical to properly differentiate the predictor functions, such as using different training subsets, initialization, architectures, and loss functions,^41^^,^^44^^,^^45^ akin to how biological circuits may encode the same stimulus with different synaptic patterns or associated valences. The second is to improve the resource efficiency of constructing multiple models, such as learning different training data in parallel.^44^^,^^45^ Beyond explicitly training separate models, implicit ensemble methods achieve diversity by reusing a single model’s intermediate states or sub-configurations.^46^^,^^47^ For example, dropout randomly deactivates units during training, generating diverse sub-models from the same network. Likewise, temporal parameter snapshots preserve model states at different training iterations, which can be averaged to emulate an ensemble without retraining. The third is to make an appropriate integration of multiple predictions, such as performing unweighted or weighted average^48^ and majority voting,^44^ reminiscent of how MBONs in *Drosophila* combine outputs of different compartments.

Unlike EL, MoE was originally proposed to alleviate interference for learning distinct data distributions, often corresponding to multiple (sub-)tasks that may compete with each other^18^^,^^49^^,^^50^ (Figure 3D). While also combining multiple individual models, MoE incorporates a routing function that dynamically assigns input data to only a few models, thereby allowing them to differentiate specific expertise (so-called experts). It determines which experts are activated for a given input, typically based on learned criteria such as data features or task identities, enabling conditional computation and specialization. This design also avoids the simultaneous use of all models during training and testing. As a result, the sparsity of model selection becomes the primary technical challenge.^51^^,^^52^ A common implementation is to learn the routing function with a SoftMax operation, and then select the top-*k* experts for input data.^18^^,^^51^^,^^52^ Some recent studies also assign different portions of the input data to each expert,^53^ or employ an unlearned routing function.^54^ Sparse MoE enjoys the theoretical foundation that learning distinct data distributions with intrinsic clustering structures can avoid collapsing into a single model and thus improve the performance.^50^

Interestingly, when considering realistic learning processes that evolve over time, many advanced applications of EL and MoE tend to incorporate hybrid characteristics from each other, but their respective strengths are greatly compromised due to mismatched assumptions about data distributions. This challenge is particularly evident in the context of continual learning, which refers to the ability to acquire and retain knowledge across sequential experiences without overwriting previously learned information. BI naturally excels at continual learning, whereas AI often exhibits catastrophic forgetting.^55^^,^^56^ In response, EL has been extended to sequentially arriving tasks by learning each task with an individual model^57^^,^^58^ (Figure 3C). This idea later developed into architecture-based methods of continual learning by constructing task-specific parameter (sub-)spaces.^55^^,^^59^^,^^60^ However, these models are tailored to distinct data distributions and no longer serve to promote generalization. In fact, the learning capacity of each task is often greatly impaired due to the constraints of available parameters and knowledge transfer.

In recent years, large-scale machine learning models initialized with massive pre-collected data (so-called foundation models) have greatly improved the adaptability of AI. They typically employ sparse MoE-based architectures, which allow scaling the model with continually collected data without much increase in computation^51^^,^^52^ (Figure 3E). Especially with large language models (LLMs), these data often have similar knowledge structures, making it difficult for MoE to differentiate specific expertise.^61^ Consequently, the routing decisions depend heavily on the token identity with minimal context relevance and task specificity.^62^ To further improve the efficiency of scaling MoEs, recent upcycling strategies perform continual training of massive expert copies,^63^ which may reinforce this issue as all experts are initialized to be the same.

## Interdisciplinary comparison of multi-modular architectures

Targeting the objective of adaptive learning (Figure 3A), the hierarchical multi-modular architecture of the *Drosophila* MB may offer an elegant combination of MoE as the first layer and EL as the second layer (Figure 5). Numerous theoretical models have been developed to explain MB learning mechanisms, exploring diverse learning rules and circuit formulations.^64^ Here, rather than re-surveying these models, we provide a complementary machine learning perspective that highlights shared algorithmic principles with MB computations. Specifically, upon perceiving an incoming experience, a sparse population of KCs is activated under a particular routing strategy.^12^ The three major types of these KCs and the multiple compartments of their axons are responsible for different memory components, which enable information processing across different time scales after learning. Their respective outputs are then dynamically integrated to guide adaptive behaviors. This hierarchy may help to realize the functions of MoE and EL individually, while interdependently, rather than mixing them into a single layer in conflict with each other. Consequently, the learning objectives of promoting generalization and alleviating interference are subtly combined together to enhance adaptability. The organizing principles of KCs, DANs, MBONs, and their particular connections further contribute to addressing the key technical challenges of MoE (i.e., sparsity) and EL (i.e., diversity, efficiency, and integration), respectively.

### Sparsity

**Figure 4:**
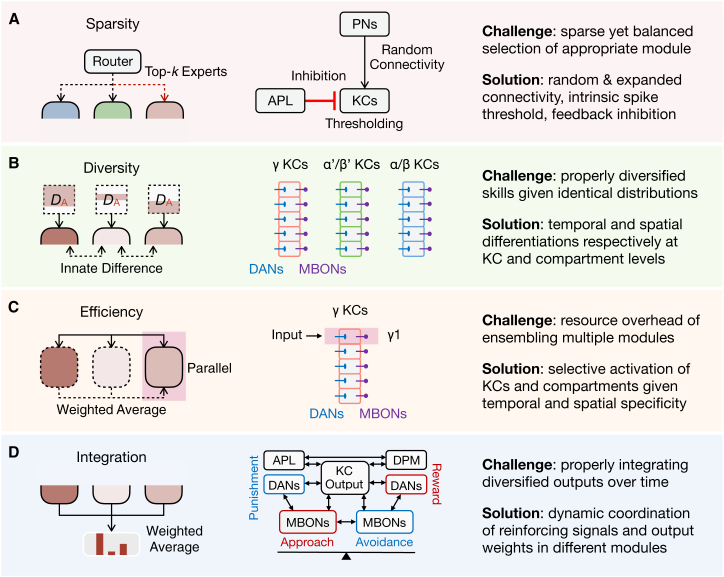
Comparative strategies for learning across multiple modules (A) Sparsity. MoE often incorporates sparsity regularization in constructing the routing function. *Drosophila* achieves sparse olfactory representations via the random convergence of PN-to-KC connectivity, the intrinsic spike threshold of KCs, and the feedback inhibition from a pair of neurons called anterior paired lateral (APL). (B) Diversity. EL often employs different parts of training data and innate differences of individual models to induce diversity. *Drosophila* exhibits diversity at levels of both KC types and their compartments. (C) Efficiency. EL often employs a data parallel or implicit ensemble to reduce resource overhead. *Drosophila* selectively activates different KCs and compartments depending on the type of learning task and different time scales after learning. (D) Integration. EL often integrates the outputs of individual models in a weighted average manner. *Drosophila* employs a dynamically coordinated approach to integrate MB outputs. APL and dorsal paired medial (DPM) are two pairs of modulatory neurons that provide feedback inhibition to the KC compartments. The compartments can be divided into two groups guiding opposite behaviors, modulated by the DANs that respond to different reinforcing signals.

The identity information of a given odor can be represented by approximately 5% KCs with a sparse activity pattern.^65^^,^^66^ This arises from the random convergence of PN-to-KC connectivity that projects neural representations from a low-dimensional space to a high-dimensional space,^67^ the intrinsic spike threshold of KCs that tends to be activated only with a combination of multiple inputs,^68^ and the feedback inhibition from a single large inhibitory neuron that attenuates the signals of KCs with relatively weak activation^69^ (Figure 4A). There is less overlap in KC response patterns for different odors and more overlap for similar odors.^70^ The sparsity of activation allows for less mutual interference between tasks associated with different odors, which may inspire designing more efficient and accurate routing functions in MoE-based architectures. For example, the high-dimensional space can make different data distributions easier to separate and recognize. The external inhibition and internal thresholding constitute a particular winner-take-all strategy that further enhances contrast and avoids redundant computation.

### Diversity

The diversity of MB is reflected at two levels, KCs and compartments (Figure 4B). In olfactory learning and memory, γ KCs, α′/β′ KCs, and α/β KCs play different roles in memory processing, from short-term to long-term.^31^^,^^71^^,^^72^ Each major type of KCs further transmits olfactory information to different downstream MBONs through its five compartments, which also receive simultaneous modulations from different DANs and thus diversify the processing of olfactory information. New evidence suggests that these three major types of KCs can be further categorized into fourteen subtypes^10^ that further diversify their functions. Such diversified processing of the same experience on spatial and temporal scales may capitalize on the benefits of both explicit and implicit ensembles. This allows previously learned and currently acquired information to be represented in different groups of modules, with different modules in each group being modulated by a “loss function” of distinct supervisory signals, thereby representing external changes as much as possible in a finite-sized model.

### Efficiency

For resource efficiency, it is preferable to select only a few modules for learning or performing specific tasks rather than using them all. The MB seems to adopt a similar principle (Figure 4C). Specifically, different types of KCs are sequentially applied in learning and remembering,^31^^,^^71^^,^^72^^,^^73^ and even different compartments of the same type of KCs are selectively engaged.^38^^,^^73^ For example, the γ1 compartment is thought to play a major role in aversive learning.^38^^,^^39^^,^^74^^,^^75^ Therefore, the selected compartments may switch dynamically over time, possibly due to their complicated interconnection patterns.^6^^,^^10^^,^^76^ Note that such efficient selection is performed at the level of KC compartments, different from the routing function that selects a sparse amount of KCs at the level of neuronal populations. These two levels of selection (i.e., the diversified multiple modules and the neural populations corresponding to an experience in each module) also suggest a promising direction for accommodating EL and MoE together, with the latter alleviating interference between experiences and the former promoting generalization via diversified information of each experience.

### Integration

**Figure 5:**
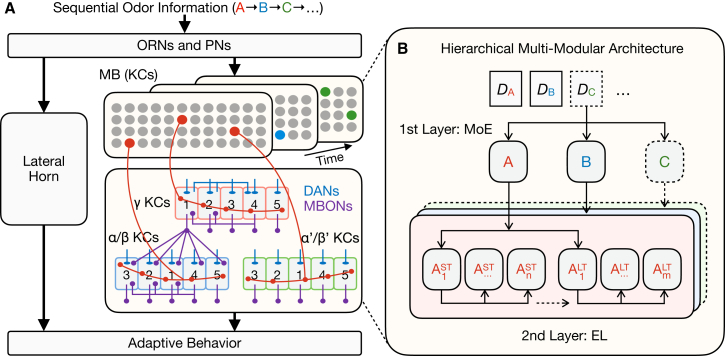
Convergent computational principles in *Drosophila* olfactory learning and machine learning (A) The *Drosophila* MB exemplifies a hierarchical, multi-modular architecture. (B) This architecture parallels a hybrid of MoE (the first layer) and EL (the second layer), combining their respective advantages. In addition, the parallel organization of LH and MB takes advantage of both innate and learned behaviors as a holistic strategy of adaptation. *D*_A_, *D*_B,_ and *D*_C_ denote the training data of tasks A, B, and C, respectively. ST and LT denote comparably short-term and long-term information after learning, respectively. n and m denote the number of modules representing diversified information. Such information corresponds to the multiple memory components of MB, performing EL at different temporal scales.

Integrating the outputs of different KC compartments is complicated and dynamic (Figures 4D and 5A). First, the five compartments of each major type of KCs can be divided into two groups guiding opposite behaviors (i.e., approach or avoidance),^38^ and modulated in a switchboard-like coordination^77^ via the DANs delivering reward or punishment signals, respectively,^7^ as well as via feedback inhibition from two pairs of neurons.^78^^,^^79^^,^^80^ Second, the five compartments of different KCs exhibit unique connection patterns, such as the recurrent loop between γ1-γ2 and γ4 compartments^6^ and the information flow from β1 to α1-α3 compartments.^6^ Third, direct connections also exist between compartments from different KCs. The output neuron of the γ1 compartment can provide inhibition to all compartments of α/β KCs.^6^ New evidence reveals that DANs can integrate both innate and learned valences of odors, mediating the circuit interactions between STM and LTM.^81^

Unlike the static, feedforward integration (e.g., weighted averaging) commonly used in EL and MoE, the MB implements a more adaptive, temporally coordinated integration strategy. Through recurrent loops and neuromodulatory signals, the MB flexibly combines information across timescales and memory types, adjusting outputs in real time based on context and internal state. In piecewise-static environments, this mechanism may functionally align with conventional EL and MoE operating on batched data. However, in dynamic or ambiguous contexts, the MB’s flexible routing and integration strategies allow more responsive and efficient adaptation. This suggests that incorporating such biologically grounded integration strategies into artificial systems may enhance robustness and context sensitivity.

## Toward more adaptive machine learning models

The above biological strategies for sparsity, diversity, efficiency, and integration offer a rich source of inspirations for advancing EL and MoE implementations. The two-layer hierarchy exemplifies how both approaches can be optimized respectively yet collaborate seamlessly, which provides a biologically informed design that promotes generalization and mitigates interference over time. The hierarchical organization of routing and integration reflects a natural division of roles within the *Drosophila* brain, as supported by recent findings showing that the MB γ lobe dynamically recruits different compartments to promote Rac1-mediated forgetting,^76^ which affects interference between sequential learning tasks.^82^^,^^83^

To facilitate understanding, we propose here a conceptual model as a concrete example (Figure 5B): it may involve two neural networks, each consisting of multiple parallel modules (i.e., sub-networks), corresponding to the STM of γ KCs and the LTM of α/β KCs. The former learns current training data (i.e., an experience), while the latter consolidates previously learned knowledge from the former. A simple implementation of their interplay, akin to memory stabilization, can be either moving averaging of network parameters or knowledge distillation of network outputs. Both processes require an intermediate network corresponding to the role of α′/β′ KCs. During training, the multiple parallel modules are diversified via different loss functions, coupled with learnable masks to sparsely activate sub-populations in each module (i.e., only the masked ones are updated) and a routing function to select which sub-population to activate. As such, MoE ensures that all sub-populations relevant to a given experience are activated, alleviating interference from unrelated experiences. Simultaneously, EL diversifies and integrates the sub-populations activated in different modules, promoting the generalization of the selected experience. Over time, these mechanisms are continually optimized to adapt to external changes. Such an AI prototype can be further evaluated with real-world benchmarks, such as online continual learning of heterogeneous tasks under blurred task boundaries.

Encouragingly, the key components of our conceptual model are grounded in both biological findings and computational implementations. For example, Dasgupta et al.^12^ proposed that the random projection, expanded dimension, and winner-take-all strategies inherent in PN-to-KC connectivity can serve as an effective routing function, validated on SIFT, GLOVE, and MNIST datasets. Shen et al.^84^ employed sparse, high-dimensional representations inspired by *Drosophila* to alleviate catastrophic forgetting. Wang et al.^59^^,^^85^ introduced an EL-based continual learning approach by modeling the five compartments of γ KCs, which induces the diversity of task expertise by selectively protecting and forgetting old memories, and integrates their outputs via a weighted average. The improvement of continual learning has been demonstrated on visual recognition tasks, such as Split CIFAR-100, and reinforcement learning tasks, such as Atari games.

Despite these advancements, many questions remain for future explorations. A key priority is to construct and empirically validate the proposed two-layer hierarchy in accommodating the respective strengths of EL and MoE. Then, it is necessary to determine the optimal number of multiple modules and their connectivity patterns, while implementing a selective ensembling strategy to ensure resource efficiency. Additionally, it is important to develop training and inference methods that allow the continually acquired information to propagate across different modules and dynamically integrate their outputs for better predictions. The bio-inspired strategies that address key technical challenges of EL or MoE could be developed individually and then incorporated into the two-layer hierarchy.

Such explorations hold promise to further enhance adaptability by addressing many long-standing problems in machine learning, such as continual learning, transfer learning, multi-task learning, and so forth. These problems align with the hybrid learning objectives of EL and MoE, namely, promoting generalization and alleviating interference continually. In particular, the MoE-based architectures have become a prevalent choice for scaling foundation models.^51^^,^^52^ Such models are usually pre-trained on a large number of general tasks, followed by the transfer learning of the pre-trained knowledge to resolve multiple specific tasks. As an elegant combination of EL and MoE, the MB-inspired model can provide a good reference for this paradigm via better ensembling pre-trained knowledge and differentiating task-specific experts. This design may also address combinatorial problems, such as continual pre-training and continual transfer learning of sequentially arriving tasks.

Notably, the learning strategy of the MB also differs significantly from that of conventional EL and MoE. The MB achieves data-efficient, online continual learning through sparse structural regularization (e.g., the fixed PN-to-KC wiring), compartment-specific dopaminergic signals as task-relevant feedback, and local synaptic plasticity at KC-to-MBON connections. In comparison, deep EL and MoE rely on global error backpropagation, explicit training-validation splits, and extensive hyperparameter tuning, typically in batch-based, offline settings. As a result, the MB enables rapid, low-resource adaptation in dynamic environments, while EL and MoE excel in scalability and task-specific performance, albeit demanding large datasets and supervision. These contrasts point to two implications. For artificial systems, integrating local learning rules, sparse activation, and neuromodulatory routing may improve efficiency and robustness. For neuroscience studies, combining behavioral perturbation with modeling may clarify how modular structure and plasticity support adaptive learning. Cross-disciplinary insights from both fields can inform the design of more generalizable, interpretable, and resource-efficient learning systems.

Moreover, the adaptability in organisms extends beyond learned behaviors to include innate behavioral responses. In *Drosophila*, the MB and LH are thought to mediate learned and innate behaviors, respectively,^22^^,^^23^ with increasing evidence of interaction between them^25^ (Figures 2A and 5A). LH neurons have been shown to influence memory retrieval,^24^^,^^25^ and MB neurons also influence innate behaviors,^86^ suggesting a dynamic integration of both. Despite significantly different architectures and scales, a functional analogy may be drawn to retrieval-augmented LLMs, where static external knowledge bases guide the output of a trainable prediction model.^87^^,^^88^ In this view, the external database resembles a form of “innate” prior knowledge that stabilizes or constrains flexible inference, analogous to how innate circuitry in the LH may modulate MB-driven memory-based behaviors. This analogy highlights the principle of combining flexible memory with stable, hard-coded guidance, a strategy that enhances adaptability in both BI and AI. Such interactions may inspire future designs that integrate retrieval and inference modules in more biologically informed ways.

Beyond the MB, several high-fidelity computational models of other *Drosophila* brain regions have provided valuable insights into adaptive behaviors and neural computation. For instance, hΔC fan-shaped body neurons integrate odor and wind cues to guide navigation, supported by a connectome-based model that reproduces behavioral outcomes.^1^^,^^89^ In the optic lobe, deep mechanistic networks constrained by connectome data accurately recapitulate ON/OFF responses and direction selectivity.^90^ A whole-brain leaky integrate-and-fire model encompassing around 125,000 neurons offers quantitative predictions of feeding and grooming circuits, validated via optogenetics.^91^ Additionally, a physics-based whole-body simulator equipped with learned controllers produces realistic locomotion, exemplifying the integration of biomechanics with neural control.^92^ These studies highlight how neurobiological and embodied constraints can inspire adaptive AI designs.

## Toward deeper understanding of biological adaptive learning

Although adaptive learning is considered to be an innate ability of BI in response to external changes, it remains poorly understood the neurobiological mechanisms of memory generalization and interference mitigation in adaptive learning. This is because a majority of current research has focused on precise memory for individual learning tasks. Indeed, biological memory is not simply a static record of past experiences but evolves to integrate new information from dynamic, uncertain environments.^93^ Therefore, the unique strengths of BI may be better demonstrated when assessing generalization and sequential learning. In this context, we compare the *Drosophila* olfactory learning system with two classical multi-modular methods of machine learning, highlighting the need for further exploration of how *Drosophila*’s hierarchical multi-modular architecture supports the objective of adaptive learning.

Notably, although researchers usually give animals the same task-cueing stimuli during memory tests as they do during learning, it is difficult to keep the internal state and external environment the same at the two stages. This implies that the precise memory performance often studied in BI already involves a degree of generalization. A recent study has shown that aligning the testing environment more closely with the learning environment can extend memory retrieval from about 1 day to over 2 weeks.^24^ Therefore, we argue that the precise memory performance studied extensively in BI is more analogous to the concept of generalization in AI (i.e., from training data to similar test data), while the concept of generalization in BI should correspond to the more difficult generalization tasks in AI (i.e., from training data to dissimilar test data).

In addition, current neuroscience insights into AI mainly focus on neural circuits and synaptic plasticity associated with sensory experiences or individual learning tasks. However, the understanding of how brain networks flexibly adapt to sequential learning tasks remains limited. The *Drosophila* brain stands out as a desirable model for addressing these gaps, given the unprecedented clarity achieved in its static architecture.^6^^,^^7^^,^^8^^,^^9^^,^^11^ A recent study has shown that the specific neurobiological mechanisms involved in learning different tasks sequentially in *Drosophila* do not affect the performance of learning a single task.^82^ This indicates the potential for future discoveries regarding the dynamic adjustments of neural circuits and synaptic plasticity that allow memory generalization and interference mitigation to be sustained. These findings point to a promising direction for uncovering the biological basis of adaptive learning. Future experiments using optogenetic tools and live imaging could selectively perturb individual MB compartments in response to sequential learning tasks while monitoring memory trace dynamics, allowing direct tests of their roles in memory protection, updating, and interference resolution.

While our interdisciplinary comparison reveals many similarities between BI and AI, it is equally important to recognize their fundamental differences. Biological systems evolve under constraints including metabolic cost, anatomical limitations, and ecological pressures. Their adaptability prioritizes robustness to environmental variability rather than optimization for task-general efficiency. In contrast, artificial systems are typically developed in controlled settings with abundant labeled data, aiming to maximize accuracy, scalability, or inference speed. These conditions rarely reflect the noisy, dynamic, and partially observable environments navigated by organisms. In fact, biological learning often proceeds without explicit supervision or clearly defined task boundaries, relying instead on context-dependent plasticity. The artificial systems, by contrast, depend on strong supervisory signals and static architectures. These divergent assumptions can lead to differing interpretations of similar mechanisms. Therefore, translating insights across domains requires the careful abstraction of core principles while remaining sensitive to the domain-specific origins and functions of those mechanisms.

## Conclusion

Despite different paces and focuses, the common goal of BI and AI in the adaptive learning of external changes allows them to complement the progress of each other. In this regard, the *Drosophila* olfactory learning system provides an excellent entry point, given a relatively clear understanding of its hierarchical multi-modular architecture. By conducting an interdisciplinary comparison between this system and two classical multi-modular methods in machine learning, we summarize the learning objective of adaptability, primary technical challenges involved, and potential strategies to address them, leading to a more comprehensive picture of adaptive learning at both functional and mechanistic levels. Such efforts contribute to the emerging field of NeuroAI,^1^ which explores how computational models of AI can contribute to a deeper understanding of brain mechanisms, and how insights from neuroscience can inspire better artificial systems. Notably, the availability of synapse-level connectomes, including the recently published full-brain reconstruction,^11^ provides unprecedented opportunities to test and refine such interdisciplinary hypotheses with anatomical fidelity.^15^

Looking forward, a key research priority is to develop and empirically validate computational models that preserve the respective strengths of EL and MoE. Further research may explore how to dynamically adjust inter-module connectivity and control resource allocation during continual learning. It is also important to test these models in real-world scenarios, such as continual pre-training and transfer learning across tasks with heterogeneous data distributions.

We recognize that drawing analogies between *Drosophila* and AI remains speculative, but we hope this perspective stimulates productive dialogue and cross-disciplinary exploration. Notably, translating these neuroscience insights into scalable artificial systems poses several challenges. First, the neurobiological mechanisms in *Drosophila* are highly specialized and optimized for specific evolutionary needs (as is the case in all animals, including humans), which may not directly generalize to complex real-world datasets. Second, biological constraints such as energy efficiency and anatomical structure differ substantially from computational constraints in artificial systems, complicating direct implementation. Finally, while the static architecture of the *Drosophila* brain is well characterized, fully linking its rich functional dynamics under naturalistic learning conditions to general computational principles remains challenging. Achieving this requires a careful balance between abstraction and biological fidelity, ultimately enabling future research to more effectively synergize neuroscience insights with algorithmic innovations to build adaptive, robust, and efficient learning systems.

### Limitations of the study

This perspective conceptually aligns biological learning and machine learning, focusing on the *Drosophila* MB and multi-modular methods. However, several limitations should be noted. First, the analogies drawn between EL, MoE, and the biological architecture are inherently speculative and abstract, given differences in design principles, learning objectives, and constraints. Second, while we highlight functional and anatomical correspondences, the biological mechanisms are context-dependent and optimized for evolutionary purposes, which may not directly translate to artificial systems. Finally, the discussion is primarily qualitative and conceptual. Future work will be needed to quantitatively test and formalize these hypotheses through computational modeling and biological experimentation.

## Acknowledgments

We thank Jun Zhou, Ning Huang, Hongwei Yan, Guanglong Sun, and Min Zhao for helpful discussions on related topics. This work was supported by the 10.13039/501100001809NSFC Projects (62406160, to L.W.), and the Fundamental Research Funds for the Central Universities, 10.13039/501100002402Sun Yat-sen University (59000-31610021, to Q. L.).

## Author contributions

L.W. and Q.L. conceived the project. L.W. wrote the content of machine learning, and Q.L. wrote the content of biological learning. Both authors wrote the interdisciplinary content and revised the paper.

## Declaration of interests

The authors declare no competing interests.
